# CREB1 regulates KPNA2 by inhibiting mir-495-3p transcription to control melanoma progression: The role of the CREB1/miR-495-3p/KPNA2 axis in melanoma progression

**DOI:** 10.1186/s12860-022-00446-1

**Published:** 2022-12-15

**Authors:** Xuerui Geng, Xiujuan Qiu, Jun Gao, Zhifan Gong, Xiaogang Zhou, Chunlei Liu, Haichao Luo

**Affiliations:** Xiangyang No 1 People’s Hospital Affiliated to Hubei University of Medicine, No.15 Jiefang Road, Fancheng district, Hubei Province 441000 Xiangyang City, China

**Keywords:** CREB1, MiR-495-3p, KPNA2, Melanoma

## Abstract

**Background:**

Melanoma is a common type of skin cancer, and its incidence is increasing gradually. Exploring melanoma pathogenesis helps to find new treatments.

**Objective:**

We aimed to explore the potential molecular mechanisms by which CREB1 regulates melanoma.

**Methods:**

TransmiR and ALGGEN were used to predict targets of CREB1 in the promoter of miR-495-3p or miR-495-3p and KPNA2, and a dual-luciferase reporter assay was performed to detect binding of CREB1 to these promoters. In addition, binding of CREB1 to the miR-495-3p promoter was confirmed by a ChIP assay. qRT‒PCR was carried out to detect mRNA levels of miR-495-3p, CREB1 and KPNA2. An EdU assay was conducted to detect cell viability. Transwell assays and flow cytometry were performed to assess cell migration and invasion and apoptosis, respectively. Moreover, factors associated with overall survival were analysed by using the Cox proportional hazards model.

**Results:**

Our results show miR-495-3p to be significantly decreased in melanoma. Additionally, miR-495-3p overexpression inhibited melanoma cell viability. CREB1 targeted miR-495-3p, and CREB1 overexpression enhanced melanoma cell viability by inhibiting miR-495-3p transcription. Moreover, miR-495-3p targeted KPNA2, and CREB1 regulated KPNA2 by inhibiting miR-495-3p transcription to enhance melanoma cell viability.

**Conclusion:**

CREB1 regulates KPNA2 by inhibiting miR-495-3p transcription to control melanoma progression. Our results indicate the molecular mechanism by which the CREB1/miR-495-3p/KPNA2 axis regulates melanoma progression.

## Introduction

Melanoma is a malignant tumour of the skin, usually rooted deep in the epidermis, and is characterized by the production of melanin [1]. In recent years, the incidence of melanoma has gradually increased, and its metastatic behaviour is considered to be the main cause of death [2]. Therefore, early identification and diagnosis are very important for the treatment of patients with melanoma. Once a tumour metastasizes, the prognosis is very poor [3]. According to the location, stage and genetic characteristics of melanoma, photodynamic therapy, surgical resection and chemotherapy combined with radiotherapy are widely used in the treatment of melanoma [4]. However, the 5-year survival rate remains low due to the highly aggressive nature of melanoma. Hence, there is an urgent need to seek new treatments and strategies.

MicroRNAs (miRNAs) are short non-coding RNAs with a length of approximately 20 bp. MiRNAs inhibit gene expression at the post-transcriptional level by binding to the 3’ untranslated region. An imbalance of miRNAs is related to tumorigenesis, proliferation, metastasis and drug resistance [5]. It has been reported that miR-495-3p dysregulation is closely related to tumour occurrence. Zhao et al. showed that miR-495-3p was down-regulated in osteosarcoma (OS). Overexpression of miR-495-3p inhibits OS metastasis by negatively regulating CTRP3, and miR-495-3p, as a tumour suppressor, is a potential biological target for OS treatment [6]. Li et al. reported that miR-495-3p is expressed at low levels in glioma tissues and cells and is involved in the regulation of glioma angiogenesis [7]. Moreover, Zhang et al. indicated that miR-495-3p is expressed at low levels in colorectal cancer (CRC) tissues and cell lines and that miR-495-3p overexpression inhibits CRC cell proliferation by targeting HMGB1 [8]. Based on the above findings, miR-495-3p plays a negative role in most tumours and is used as a therapeutic target. However, there are few reports about miR-495-3p in melanoma, and the potential molecular mechanism has not been elucidated.

As a transcription factor involved in metabolism and DNA repair, cAMP-responsive element-binding protein-1 (CREB1) has been proven to play a carcinogenic role in various cancers. In addition, CREB1 can induce transcription of miRNA. Zhao et al. reported that CREB1 induces miR-1204 overexpression and promotes the malignant phenotype of glioblastoma [9]. Li et al. showed that CREB1 activates miR-433 expression and that CREB1/miR-433 plays a key regulatory role in CRC progression [10]. CREB1 modulates cancer progression through transcriptional regulation of microRNA and is a promising molecular target. At the beginning of our study, we detected a binding site for CREB1 in miR-495-3p based on ALGGEN prediction, and we speculated that CREB1 may regulate miR-495-3p expression. However, the specific mechanism of the CREB1/miR-495-3p axis in melanoma progression is unclear.

Karyopherin α2 (KPNA2) is one of the seven members of the nuclide alpha protein family and plays a key role in nucleocytoplasmic transport [11]. KPNA2 may mediate nuclear transport of tumour suppressors, and its up-regulation is closely related to all kinds of malignant tumours. Specifically, KPNA2 promotes tumour formation and progression through cell differentiation, proliferation and apoptosis [12, 13]. Initially, the binding sequence between miR-495-3p and KPNA2 was predicted through the StarBase website. However, whether miR-495-3p is involved in melanoma progression by targeting KPNA2 remains unclear.

In summary, we hypothesized that the transcription factor CREB1 increases KPNA2 expression by inhibiting miR-495-3p transcription, which in turn promotes the development and metastasis of melanoma cells. We aimed to elucidate the underlying mechanism of the CREB1/miR-495-3p/KPNA2 axis in melanoma progression. Our research provides a new direction for melanoma treatment.

## Materials and methods

### Specimens

From January 2021 to February 2022, after receiving written informed consent, 30 patients at Xiangyang First People’s Hospital Affiliated to Hubei Medical College with melanoma and surgical tumour samples and adjacent non-tumour tissues were recruited. The selected patients did not receive chemotherapy /or radiotherapy. This study was approved by the Ethics Committee of Xiangyang First People’s Hospital Affiliated to Hubei Medical College, and written informed consent was obtained from all patients.

### Cell lines and culture

Procell Life Science and Technology Co. Ltd. (Wuhan, China) provided the HEMa-LP cells and melanoma cell lines, including A375, A2058, B16, and MUM2B cells used in this study. The cells were cultured in Dulbecco’s modified Eagle medium containing 1% penicillin‒streptomycin solution (P/S) and 10% FBS (DEME, Gibco, New York). Cells were cultured in a humidified incubator at 37 °C and 5% carbon dioxide.

### Cell transfection

GeneChem (Shanghai, China) synthesized miR-495-3p mimics and its negative control NC mimics.

The full-length sequences of CREB1 and KPNA2 were obtained by POLYMERase chain reaction and cloned into the eukaryotic expression vector PC DNA3.1 for efficient expression of CREB1 and KPNA2. These plasmids (50 nM) were introduced into A375 and B16 cells using a liposome™3000 transfection agent (Invitrogen, California, USA).

### qRT‒PCR

Total RNA of A375 and B16 cells was extracted with TRIzol reagent (Invitrogen), and PrimeScript RT Reagent Kit (Invitrogen) was used to reverse transcribe 1 µg of total RNA to complementary DNA (cDNA) in a final volume of 10 µL. MiRNAs were collected by mirVana microRNA Isolation kits (Invitrogen), and miR-495-3p levels were detected by using the TaqMan microRNA assay kit (Invitrogen). U6 RNA served as an endogenous control. Subsequently, CREB1 and KPNA2 gene levels were quantified by qRT‒PCR. GAPDH was used as the endogenous control for data analysis. The change in mRNA level was calculated by the 2^−ΔΔCT^ method. The primers used are shown in Table 1.

**Table 1:** Primer sequences

Primer name	Primer sequences
F- miR-495-3p	5′- ACAAACATGGTGCACTTC - 3′
R- miR-495-3p	5′- GAACATGTCTGCGTATCTC - 3′
F- CREB1	5′- GACCACTGATGGACAGCAGATC - 3′
R- CREB1	5′- GAGGATGCCATAACAACTCCAGG -3′
F- KPNA2	5′- CTGTTGGCTCTCCTTGCAGTTC - 3′
R- KPNA2	5′- GCAGGATTCTTGTTGCGGCAAAG - 3′
F- U6	5′- CTCGCTTCGGCAGCACA - 3′
R- U6	5′- AACGCTTCACGAATTTGCGT - 3′
F- GAPDH	5′- GTCTCCTCTGACTTCAACAGCG - 3′
R- GAPDH	5′- ACCACCCTGTTGCTGTAGCCAA - 3′

### EdU assay

A375 and B16 cells were added to a 96-well plate at 5 × 10^4^ cells/well and incubated at 37 °C for 12 h. An EdU staining kit (Guangzhou RiboBio, China) was used for staining. Afterwards, EdU-labelled cells were analysed using a MoFlo Astrios (Beckman Coulter, Brea, CA, USA).

### Transwell assay

The migration and invasion abilities of A375 and B16 cells were detected by Transwell assays. Cells (1 × 10^5^/well) were added to the upper cavity of the inlay, and 600 µL culture medium containing 20% foetal bovine serum was added to the lower cavity. After culturing for 30 min, the migration and invasion of the cells stained with crystal violet were observed by microscopy.

### Flow cytometry

The apoptosis rate was detected using an Annexin V-FITC/PI apoptosis detection kit (Beyotime, Nanjing, China, C1062S) based on previous research [14]. In detail, after exposure of 1.0 × 10^6^ cells to 10 mL binding buffer and 1.25 mL Annexin V-fluorescein isothiocyanate, cells were incubated at room temperature for 15 min in the dark. Then, the cell suspension was centrifuged at 1000 ×g for 5 min, and the supernatant was removed; the cells were resuspended in 0.5 mL ice-cold 1× binding buffer. Finally, the cells were incubated with 10 mL propidium iodide and transferred to a fluorescent activated cell sorter tube (Fahrenheit, Munich, Germany). Fluorescence data analysis was performed with WinMDI 2.8 software.

### Dual-luciferase reporter assay

Potential binding sites for CREB1 in the promoter of miR-495-3p, miR-495-3p and KPNA2 were predicted by using StarBase bioinformatics software. Plasmid construction and luciferase activity assays were performed as previously reported [5].

### CHIP assay

Chips were analysed according to the instructions of EZ-Chiptm Chromatin Immunoprecipitation Kit (Sigma‒Aldrich, St. Louis, Missouri, USA). Chromatin fragments of 200 ~ 500 bp were immunoprecipitated with a specific antibody or negative control antibody. DNA released from the protein‒DNA complex was collected and purified with magnetic beads for RT‒qPCR analysis.

#### Statistical analysis

In this study, a mean ± standard deviation (SD) represents data from three independent trials. The *t test* was used for comparisons between two groups, and Tukey’s multiple comparison test was used for comparisons between multiple groups. *P* < 0.05 indicated a statistically significant difference.

## Results

### MiR-495-3p was significantly down-regulated in melanoma

**Fig. 1:**
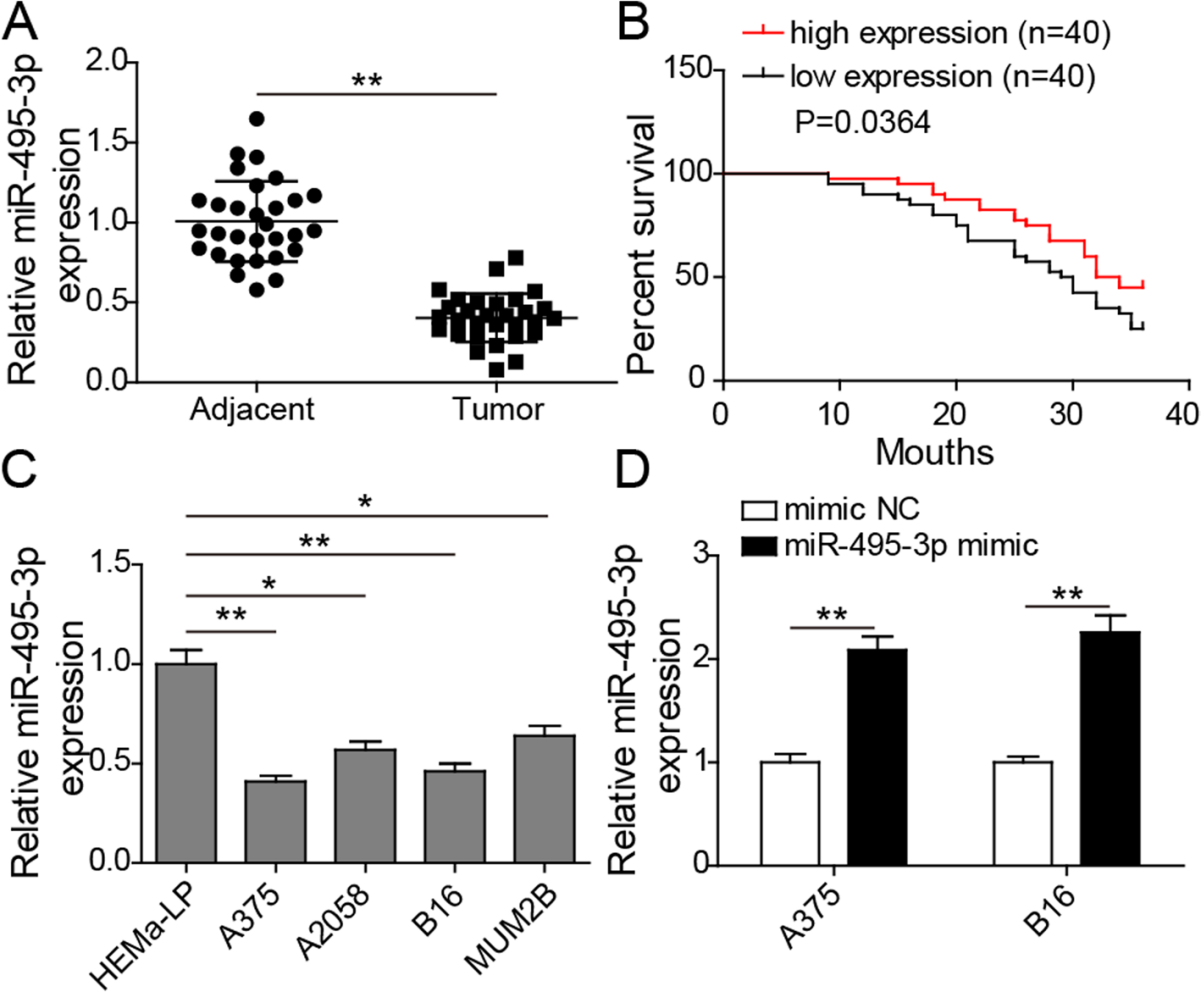
MiR-495-3p was significantly down-regulated in melanoma. **A** qRT‒PCR detected miR-495-3p levels in melanoma tissues. **B** Analysis of the relationship between miR-495-3p level and survival of patients with melanoma. **C** qRT‒PCR detected miR-495-3p levels in melanoma cells, including A375, A2058, B16, and MUM2B cells. HEMa-LP cells served as the negative control. **D** qRT‒PCR detected miR-495-3p levels after A375 and B16 cells were transfected with miR-495-3p mimics. The mean ± standard deviation represents data from three independent trials (*n* = 3). *p < 0.05, ** *p* < 0.01, *** *p* < 0.001

To explore the potential molecular mechanism of miR-495-3p in melanoma, we analysed the level of miR-495-3p in melanoma cells and tissues. Analysis showed that miR-495-3p was decreased in melanoma tissues; miR-495-3p levels correlated negatively with the survival of patients with melanoma (Fig. 1 A and B). Similarly, miR-495-3p was decreased in melanoma cells, especially in A375 and B16 cells (Fig. 1 C). Therefore, A375 and B16 cells were used for subsequent functional studies. qRT‒PCR analysis further showed that miR-495-3p was overexpressed after A375 and B16 cells were transfected with miR-495-3p mimics (Fig. 1D).

### MiR-495-3p overexpression inhibited melanoma cell viability

**Fig. 2:**
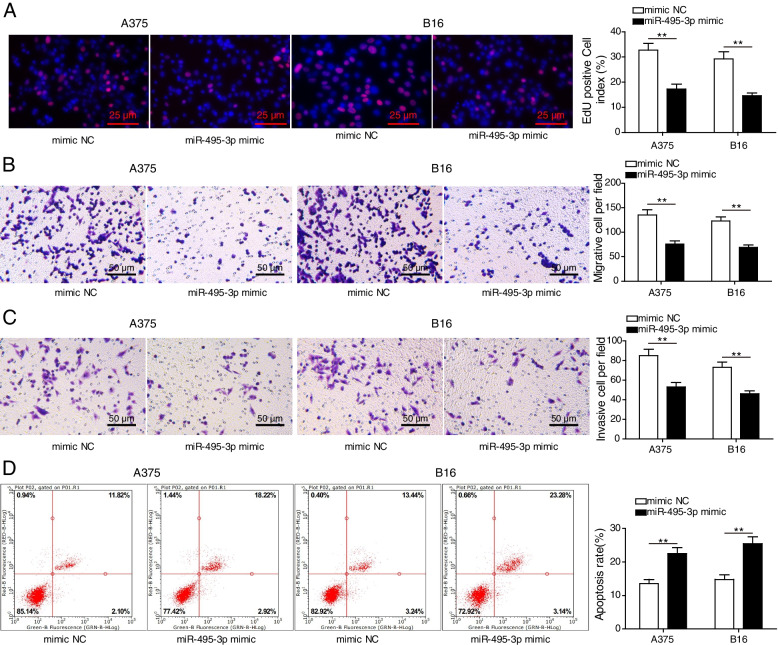
MiR-495-3p overexpression inhibited melanoma cell viability. **A** EdU assays detected A375 and B16 cell viability. **B-C** Transwell assays detected A375 and B16 cell migration and invasion. **D** Flow cytometry detected apoptosis. The mean ± standard deviation represents data from three independent trials (*n* = 3). *p < 0.05, ** *p* < 0.01, *** *p* < 0.001 (*n* = 3)

To further investigate the underlying molecular mechanism by which miR-495-3p regulates melanoma progression, melanoma cell bioactivities were analysed after A375 and B16 cells were transfected with miR-495-3p mimics. EdU assay analysis indicated significantly decreased viability of miR-495-3p-overexpressing A375 and B16 cells (Fig. 2 A). Consistently, Transwell assay analysis showed that the migration and invasion abilities of miR-495-3p-overexpressing A375 and B16 cells were significantly decreased (Fig. 2B, C). Conversely, miR-495-3p-overexpressing A375 and B16 cells had increased apoptosis rates (Fig. 2D). In summary, miR-495-3p overexpression inhibited melanoma cell viability.

### CREB1 targeted the promoter of miR-495-3p

**Fig. 3:**
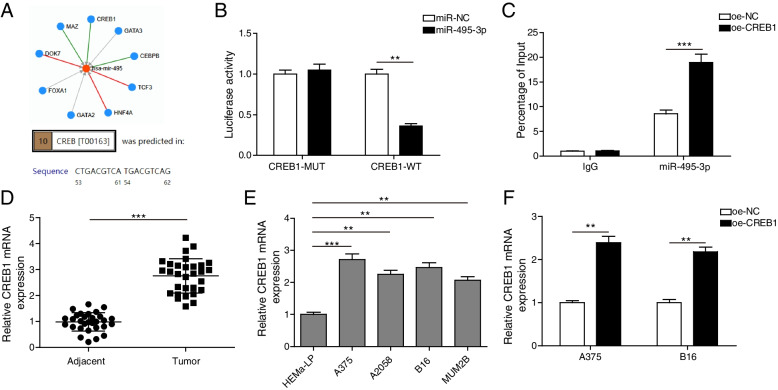
CREB1 targeted the promoter of miR-495-3p. **A** TransmiR and ALGGEN were used to predict transcription factors regulating miR-495-3p. **B** A dual-luciferase reporter assay detected binding of CREB1 to the promoter of miR-495-3p. **C** Binding between CREB1 and the promoter of miR-495-3p was confirmed by the ChIP assay. **D** qRT‒PCR detected CREB1 levels in melanoma tissues. **E** qRT‒PCR detected CREB1 levels in melanoma cells, including A375, A2058, B16, and MUM2B cells. HEMa-LP cells served as the negative control. **F** qRT‒PCR detected CREB1 levels after A375 and B16 cells were transfected with oe-CREB1. The mean ± standard deviation represents data from three independent trials (*n* = 3). *p < 0.05, ** *p* < 0.01, *** *p* < 0.001 (*n* = 3)

First, TransmiR and ALGGEN were used to predict transcription factors regulating miR-495-3p. The results showed binding sites for CREB1 in the promoter sequence of miR-495-3p (Fig. 3 A). Subsequently, dual-luciferase reporter assay analysis revealed that co-transfection of oe-CREB1 inhibited the luciferase activity of the miR-495-3p-WT reporter gene but did not change the luciferase activity of the miR-495-3p-MUT reporter gene (Fig. 3B). The ChIP assay also showed the physical interaction between CREB1 and the promoter of miR-495-3p in melanoma cells (Fig. 3 C). Moreover, qRT‒PCR analysis showed that CREB1 was significantly up-regulated in melanoma tissues and cells, especially in A375 and B16 cells (Fig. 3D and E). Therefore, A375 and B16 cells were used for subsequent functional studies. In addition, CREB1 mRNA levels were significantly up-regulated after A375 and B16 cells were transfected with oe-CREB1 (Fig. 3 F). Overall, CREB1 targeted the promoter of miR-495-3p, and CREB1 was up-regulated in melanoma tissues and cells.

### CREB1 overexpression enhanced melanoma cell viability by inhibiting mir-495-3p transcription

**Fig. 4:**
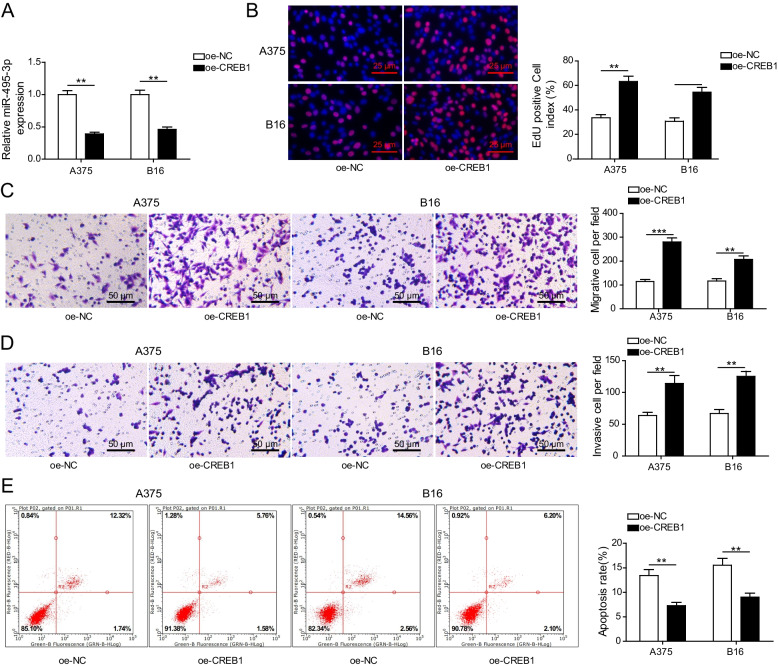
CREB1 overexpression enhanced melanoma cell viability by inhibiting miR-495-3p transcription. **A** qRT‒PCR detected miR-495-3p levels after A375 and B16 cells were transfected with oe-CREB1. **B** EdU assays detected A375 and B16 cell viability. **C-D** Transwell assays detected A375 and B16 cell migration and invasion. **E** Flow cytometry detected cell apoptosis. The mean ± standard deviation represents data from three independent trials (*n* = 3). *p < 0.05, ** *p* < 0.01, *** *p* < 0.001 (*n* = 3)

To explore whether CREB1 affects melanoma progression by regulating miR-495-3p transcription, melanoma cell bioactivities were analysed after A375 and B16 cells were transfected with oe-CREB1. First, qRT‒PCR analysis showed that miR-495-3p was significantly down-regulated after A375 and B16 cells were transfected with oe-CREB1 (Fig. 4 A); EdU assay analysis showed that the viability of CREB1-overexpressing A375 and B16 cells was significantly increased (Fig. 4B). Similarly, the abilities of CREB1-overexpressing A375 and B16 cells to migrate and invade were significantly elevated (Fig. 4 C and D). Conversely, flow cytometry analysis indicated that CREB1-overexpressing A375 and B16 cell apoptosis was significantly decreased (Fig. 4E). In conclusion, CREB1 overexpression enhanced melanoma cell viability by inhibiting miR-495-3p transcription.

### Mir-495-3p targeted KPNA2

**Fig. 5:**
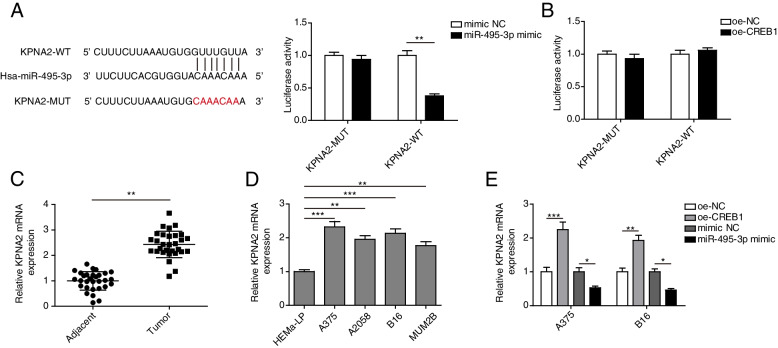
miR-495-3p targeted KPNA2. **A** Bioinformatics analysis predicted the relationship between miR-495-3p and KPNA2, and a dual-luciferase reporter assay detected binding between miR-495-3p and KPNA2. **B** A dual-luciferase reporter assay detected binding of CREB1 to KPNA2. **C** qRT‒PCR detected KPNA2 levels in melanoma tissues. **D** qRT‒PCR detected KPNA2 levels in melanoma cells, including A375, A2058, B16, and MUM2B cells. HEMa-LP cells served as the negative control. **E** qRT‒PCR detected KPNA2 levels after A375 and B16 cells were transfected with oe-CREB1 and miR-495-3p mimics. The mean ± standard deviation represents data from three independent trials (*n* = 3). *p < 0.05, ** *p* < 0.01, *** *p* < 0.001 (*n* = 3)

Bioinformatics analysis revealed binding sites in miR-495-3p and KPNA2, which was confirmed by a dual-luciferase reporter assay. In detail, co-transfection of miR-495-3p mimics inhibited the luciferase activity of the CREB1-WT reporter gene but did not change that of the CREB1-MUT reporter gene (Fig. 5 A), indicating that miR-495-3p targeted KPNA2. In contrast, dual-luciferase reporter assay analysis showed no targeting relationship between CREB1 and KPNA2 (Fig. 5B). Subsequently, KPNA2 expression was significantly decreased in melanoma tissues and cells, especially in A375 and B16 cells (Fig. 5 C and D). Furthermore, CREB1 overexpression promoted KPNA2 expression, but the upward trend was reversed by miR-495-3p overexpression (Fig. 5E). Therefore, we believe that miR-495-3p targets KPNA2.

### CREB1 regulated KPNA2 by inhibiting mir-495-3p transcription to enhance melanoma cell viability

**Fig. 6:**
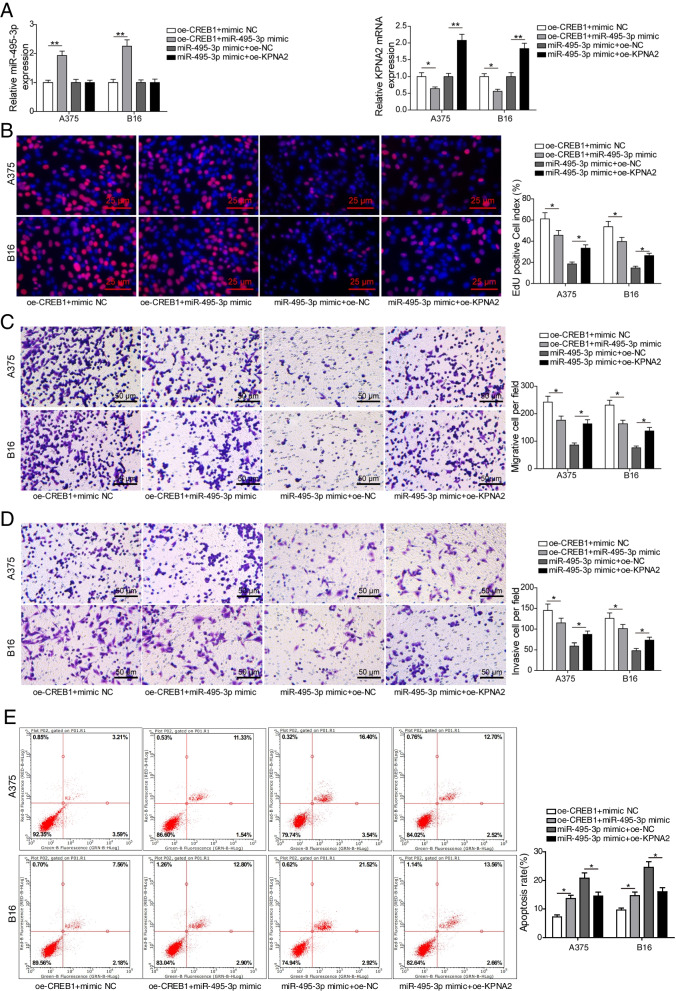
CREB1 regulated KPNA2 by inhibiting miR-495-3p transcription to enhance melanoma cell viability. **A** miR-495-3p and KPNA2 mRNA levels were detected after CREB1-overexpressing A375 and B16 cells were transfected with miR-495-3p mimics or miR-495-3p-overexpressing A375 and B16 cells were transfected with oe-KPNA2. **B** EdU assays detected A375 and B16 cell viability. **C-D** Transwell assays detected A375 and B16 cell migration and invasion. **E** Flow cytometry detected apoptosis. The mean ± standard deviation represents data from three independent trials (*n* = 3). *p < 0.05, ** *p* < 0.01, *** *p* < 0.001 (*n* = 3)

MiR-495-3p and KPNA2 mRNA levels were detected after CREB1-overexpressing A375 and B16 cells were transfected with miR-495-3p mimics or miR-495-3p-overexpressing A375 and B16 cells were transfected with oe-KPNA2. The results showed an increase in miR-495-3p mRNA by miR-495-3p overexpression, whereas KPNA2 overexpression had no effect on miR-495-3p expression. Conversely, KPNA2 mRNA levels were down-regulated by miR-495-3p overexpression, though the impact of miR-495-3p overexpression was subsequently recovered by KPNA2 overexpression (Fig. 6 A). Next, EdU assay analysis showed that the CREB1 overexpression-induced increase in A375 and B16 cell viability was inhibited by miR-495-3p overexpression and that the impact of miR-459-3p overexpression was subsequently recovered by KPNA2 overexpression (Fig. 6B). Similar results were observed for A375 and B16 cell migration and invasion (Fig. 6 C and D). According to flow cytometry analysis, the CREB1 overexpression-decreased apoptosis in A375 and B16 cells was rescued by miR-495-3p overexpression, but KPNA2 overexpression antagonized the effect of miR-495-3p overexpression (Fig. 6E). Therefore, we conclude that CREB1 regulated KPNA2 by inhibiting miR-495-3p transcription to enhance melanoma cell viability.

## Discussion

The incidence of melanoma is increasing yearly. Elucidating the pathogenesis of melanoma is beneficial for finding new treatment methods [15, 16]. We mainly clarify the role of the CREB1/miR-495-3p/KPNA2 axis in melanoma. Our findings illustrate that CREB1 regulates KPNA2 by inhibiting miR-495-3p transcription to control melanoma progression. Our study demonstrates for the first time that CREB1 participates in the regulation of melanoma progression through the miR-495-3p/KPNA2 axis.

MiR-495-3p is a key regulator of tumorigenesis and malignant progression. It has been reported that miR-495-3p is up-regulated in various tumour tissues, including melanoma [17]. Our findings reveal that miR-495-3p was significantly down-regulated in melanoma and that miR-495-3p overexpression inhibited melanoma cell viability. These findings suggest that miR-495-3p acts as a tumour suppressor involved in the negative regulation of melanoma progression. MiRNAs can be transcriptionally activated by certain transcription factors [18]. CREB1 has been shown to be involved in the regulation of various malignant tumours, including melanoma. The specific mechanism may be CREB1 transcriptional regulation of miRNAs, thus promoting tumour development. Liu et al. showed that CREB1 promotes the malignant behaviour of hepatocellular carcinoma cells via transcriptional regulation of miR-922 [19]. Yan et al. reported that CREB1 induces miR-489 to promote CRC progression [20]. Our findings show that CREB1 targets the promoter of miR-495-3p. Moreover, CREB1 was down-regulated in melanoma tissues and cell lines, and CREB1 overexpression enhanced melanoma cell viability by inhibiting miR-495-3p transcription. These findings suggest that as a carcinogen involved in regulating melanin progression, CREB1 may serve as a therapeutic target for melanoma. Our study is the first to demonstrate that CREB1 is involved in the regulation of melanoma progression through transcriptional control of miR-495-3p. Increasing evidence shows that miRNAs regulate target genes involved in tumorigenesis and melanoma development. MiR-200a has been proven to inhibit CDK6 expression in metastatic melanoma cells, which is an important factor promoting tumour development [21]. Guo et al. reported that miR-18a-5p induces proliferation and inhibited apoptosis of melanoma cells by targeting EPHA7 [22]. Fen et al. showed that miR-548b inhibits the growth and metastasis of melanoma by negatively regulating HMGB1 [23]. Our results indicate that miR-495-3p targets KPNA2 in melanoma. KPNA2 is a kind of connexin that is a member of the α protein family of beneficial nuclear proteins and plays a key role in the process of nucleocytoplasmic transport [24]. Studies have shown that KPNA2 is a potential biomarker for a variety of cancers [25, 26]. Our findings show that KPNA2 is highly expressed in melanoma tissues and cell lines and that CREB1 overexpression promotes KPNA2 expression. Based on the above results, we believe that CREB1 promotes KPNA2 expression in melanoma cells by inhibiting miR-495-3p transcription. Moreover, functional experiments further showed that CREB1 regulates KPNA2 by inhibiting miR-495-3p transcription to enhance melanoma cell viability.

In summary, our research illustrates that CREB1 regulates KPNA2 by inhibiting miR-495-3p transcription to regulate melanoma progression. Our findings highlight the specific molecular mechanism by which the CREB1/miR-495-3p/KPNA2 axis regulates melanoma progression. This study on the function of the CREB1/miR-495-3p/KPNA2 axis in the development and metastasis of melanoma provides new insight into the pathogenesis of melanoma and a promising method for the treatment of melanoma.

## Data Availability

The raw data supporting the conclusions of this manuscript will be made available by the corresponding author(E-mail:liuchunlei5751@126.com), without undue reservation, to any qualified researcher.
